# Overlapping of Primary Biliary Cirrhosis and Small Duct Primary Sclerosing Cholangitis: First Case Report

**DOI:** 10.4021/jocmr1060w

**Published:** 2012-11-11

**Authors:** Elze Maria Gomes Oliveira, Patricia Marinho Oliveira, Vitoria Becker, Alessandra Dellavance, Luis Eduardo Coelho Andrade, Valeria Lanzoni, Antonio Eduardo Benedito Silva, Maria Lucia Gomes Ferraz

**Affiliations:** a Division of Gastroenterology, Federal University of Sao Paulo, Sao Paulo, Brazil; b Fleury Medicine and Health, Sao Paulo, Brasil; c Division of Pathology, Federal University of Sao Paulo, Sao Paulo, Brazil

**Keywords:** Anti-mitochondrial antibodies, Autoimmune liver disease, Antinuclear antibodies, Primary biliary cirrhosis, Small duct primary sclerosing cholangitis, Overlapping syndromes

## Abstract

Primary biliary cirrhosis (PBC) and primary sclerosing cholangitis (PSC) are both autoimmune cholestatic liver disease and the association of these two conditions in the same patient is very rare. We report the case of a female patient presenting with a cholestatic liver disease and a panel of autoantibodies specific for PBC, including antibodies to mitochondrial E2-pyruvate dehydrogenase, gp-210 and Sp-100. Beside these findings, the liver biopsy revealed concentric fibrosis of small biliary ducts and the magnetic resonance cholangiography presented no abnormal findings. Diagnosis of small duct PSC/PBC overlapping was done. No description of this association was found in the literature. Clinical and serological features of this unusual finding are discussed.

## Introduction

Although the etiology of primary biliary cirrhosis (PBC), primary sclerosing cholangitis (PSC) and autoimmune hepatitis (AIH) remains unknown, there are several case reports of association of these hepatic autoimmune conditions in the same patient [1-4]. The expression “overlapping syndrome” has been used to describe forms of auto-immune disease, generally AIH/PBC or AIH/PSC, that present typical characteristics of more than one condition in the same patient, occurring simultaneously or sequentially, and sometimes migrating from one to another clinical presentation [3-6]. However, overlapping between PBC/PSC is much less described.

PBC is mostly prevalent among women, causing destruction of biliary ducts, resulting in progressive ductopenia and cirrhosis. AMA is considered a specific biomarker of PBC and some authors describe it as the serologic signature of the disease [7, 8]. PSC, in its turn, is also a chronic cholestatic liver disease of unknown etiology, typically marked by progressive inflammation and concentric fibrosis of intra- or extra-hepatic biliary ducts, causing cirrhosis, liver failure and high incidence of cholangiocarcinoma [9-11]. So far, there are no specific serological markers for PSC and AMA is virtually absent in PSC patients [12-14].

To our knowledge, there are only five PBC/PSC overlapping cases reported in the literature, none of them corresponding to small biliary duct PSC [15-18].

Here we describe a patient with clinical, biochemical and serological markers of PBC, whose biopsy was compatible with small duct PSC.

## Case Report

A 48-year-old woman was referred to the hepatologist in order to investigate elevated levels of liver enzymes. Except for a treated systemic hypertension, mild obesity and a ten-year irregular use of amfepramone, she had no remarkable medical history. She also had no familiar history of liver disease or alcohol consumption. Physical examination revealed only mild hepatomegaly.

**Figure 1:**
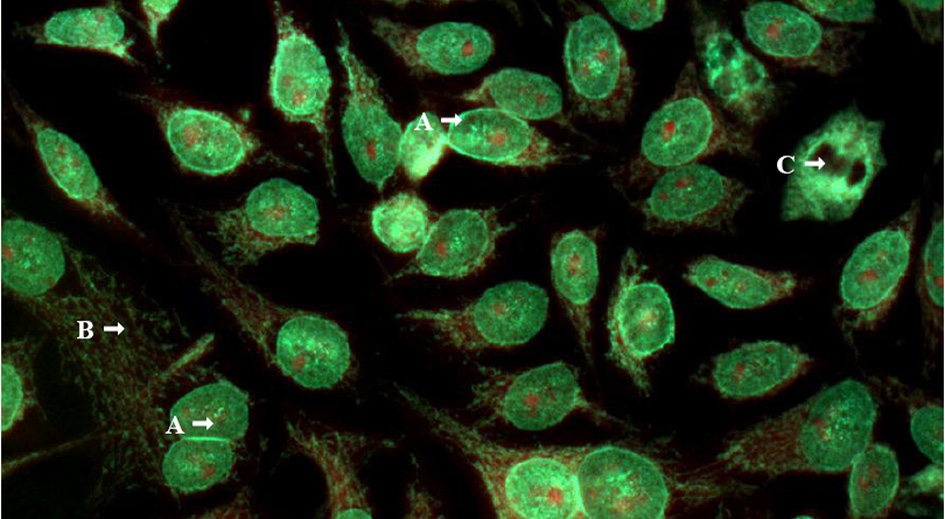
Indirect immunofluorescence on HEp-2 cells (Bion Interprise Ltd) with human serum diluted 1/80. (A) Pattern nuclear envelope; (B) and cytoplasmic discrete speckled pattern, suggestive of antimitocondrial positivity; (C) Chromosome metaphase plate negative. Magnification x 400.

Laboratory tests are summarized in Table 1. She presented a positive antinuclear antibody (ANA) test (titer > 1/640) with a rim-like membranous and cytoplasmic speckled pattern, suggestive of anti-mitochondrial positivity (Fig. 1). These ANA patterns are known to be associated to antibodies to gp210 and mitocondrial antigens, respectively. Indirect immunofluorescence (IIF) tests for anti-smooth muscle antibodies (SMA), anti-liver/kidney microsome 1 (LKM-1), and neutrophil cytoplasm antigens (ANCA) were negative. IIF-AMA on in-house rodent tissue preparations was performed as previously described [19] and was positive at 1/160. Anti-pyruvate dehydrogenase antibodies (anti-M2 fraction) were positive and detected by enzyme immunosorbent assay (ELISA-Orgentec, Mainz, Germany). There was also reactivity for anti-M2-E3 BPO, anti-gp210 and anti-Sp-100 (Euroline profile, Euroimmun, Lubeck, Germany).

**Table 1:** Case Report: Laboratory Findings

	29/04/04	28/05/04	05/06/06	31/08/06	31/08/06
AST (UI/mL)	98 (45)	79( 45)	42 (45)	116 (45)	26 (45)
ALT (UI/mL)	125 (35)	68 (35)	38 (35)	167 (35)	14 (35)
GGT (UI/mL)	227 (48)	249 (48)	408 (38)	383 (38)	12 (38)
AP (UI/mL)		472 (147)	211 (147)	468 (147)	83 (147)
Total Bilirub (mg/dL)		0.41	0.59		
Dir. Bilirub (mg/dL)		0.06	0.17		
INR			1.0		
Albumin (g/L)		4.5	4.3		
Gamma-glob (g/L)			1.6		
Total cholesterol (mg/dL)	177				
HDL -C (mg/dL)	52				
Triglycerides (mg/dL)	116				
IgM (mg/dL)		183			
IgG (mg/dL)		1460			
HBsAg	Neg				
Anti-HBc	Neg				
anti-HCV	Neg				
ANA	Pos				
anti-SMA	Neg				
anti-LKM-1	Neg				
anti-SLA	Neg				
p-ANCA	Neg				
AMA M2	Pos				
Anti-Sp100	Pos				
Anti-gp210	Pos				

HBsAg, hepatitis B surface antigen; Anti-HBc, antibody against core antigen of hepatitis B virus; anti-HCV, antibody against hepatitis C virus; ANA: anti-nuclear antibody; SMA: Smooth muscle antibodies; Anti-LKM: Liver-kidney microsomal antibodies; Anti-SLA/LP: Antibodies against soluble liver antigen/liver pancreas; pANCA: Perinuclear antineutrophil cytoplasmic antibodies. AMA M2: Antibodies to mitochondrial E2-pyruvate dehydrogenase; Anti-Sp100: Antibodies to Sp100; Anti-gp210: Antibodies to gp210.

**Figure 2:**
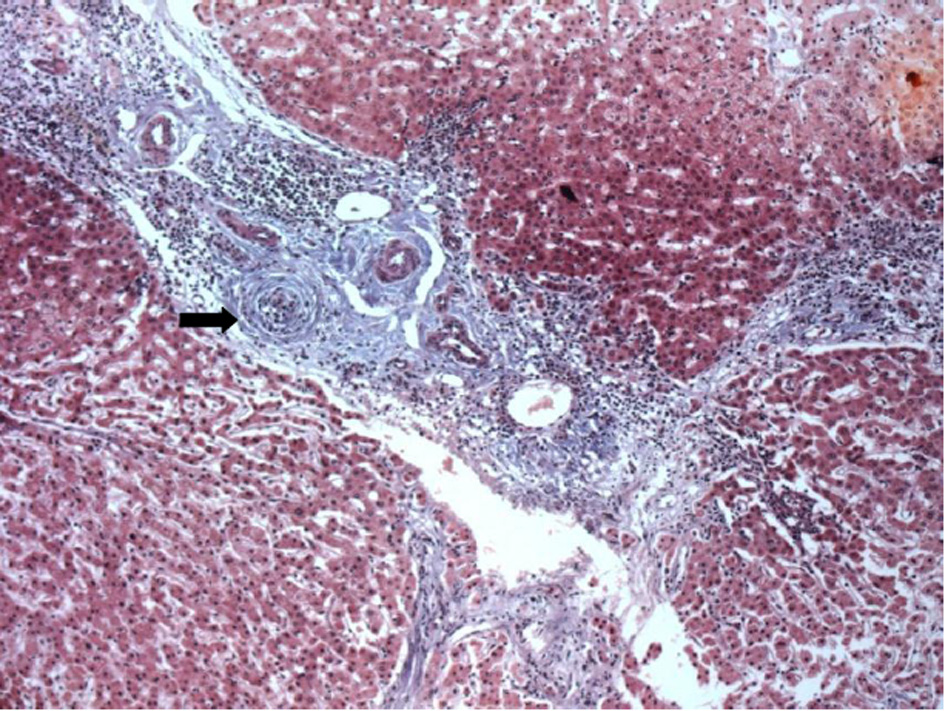
Portal fibrosis with portal-portal linking septa, and complete obliteration of bile duct which is replaced by dense fibrous whorls (Arrow). H and E, 40 x.

**Figure 3:**
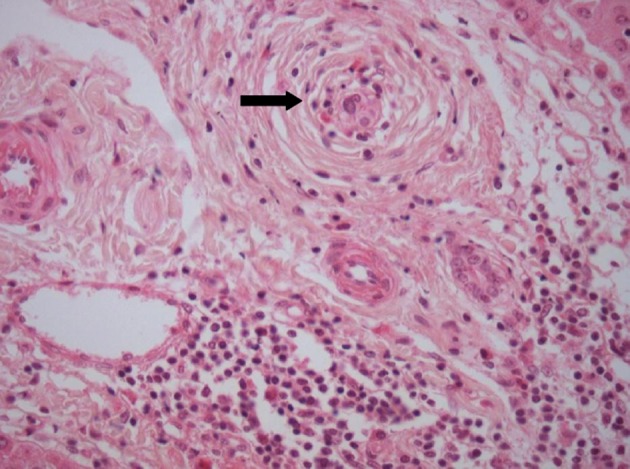
Small bile duct showing an “onion-skin” type of periductal fibrosis. H and E, 40 x.

Liver biopsy revealed bridging portal fibrosis, lymphomononuclear infiltrate with lymphocytic interface hepatitis and marginal ductular reaction. Surprisingly, some of the portal tracts revealed small biliary ducts with concentric fibrosis (“onion skin” type) with duct obliteration (Fig. 2, 3). Magnetic resonance cholangiography was then performed, with normal findings. Diagnosis of small duct PSC was done, and she was treated with ursodeoxycholic acid (UDCA) with progressive normalization of liver enzymes within 4 months.

## Discussion

The classically described overlapping syndrome is characterized by diagnosis of more than one form of autoimmune liver disease in the same patient [1-5]. If not correctly diagnosed and appropriately treated, its course can be more aggressive as compared to isolated forms [20, 21]. It is a matter of discussion if overlapping syndromes are isolated entities or different manifestations of a unique autoimmune liver disease [1, 2, 6].

Overlapping between PBC/AIH, AMA-negative PBC/AIH, and PSC/AIH, have been related in different series [3, 5, 22-24]. However, few cases of overlapping between PBC/PSC have been reported [15-18]. As both PBC and PSC are cholestatic diseases presenting with increased AP and GGT, overlapping of these two conditions could occasionally not be perceived.

The patient herein reported presented an unremarkable clinical picture represented by the incidental finding of biochemical evidence of cholestatic disease, which might be suitable to both PBC and PSC. The serologic investigation initially performed was vigorously suggestive of PBC, with a positive a panel of autoantibodies specific for this disease, including antibodies to mitochondrial E2-pyruvate dehydrogenase (AMA M2), gp-210 and Sp-100.

AMA may be considered one of the most useful parameters for the diagnosis of cholestatic liver diseases, since AMA is virtually absent in PSC patients compared to over 95% prevalence in PBC [7, 12-14, 25]. Other autoantibodies, such as anti-gp-210 and anti-Sp-100, are also specifically associated with PBC [26]. Anti-gp-210 has been associated to a more aggressive course of the disease [27, 28] whereas anti-Sp-100 is considered highly specific (specificity of 95%), despite its low sensitivity (around 30%) [27-29]. These PBC-associated autoantibodies elicit peculiar ANA patterns, such as the cytoplasmic reticule-like speckled pattern (associated with AMA) [30], the multiple nuclear dots pattern (associated to anti-Sp100 antibodies), the rim-like membranous pattern (linked to positivity to anti-gp210 antibodies), and the centromeric pattern [26-28].

Despite all these evidences for the diagnosis of PBC in the present case, the histological examination was not characteristic of the disease; instead, it showed typical onion-skin lesions in biliary ducts, with concentric fibrosis, compatible with PSC, a non-expected finding in face of the serological findings.

The diagnosis of PSC is confirmed by the demonstration of typical lesions of the biliary tree, either by histological analysis or by imaging techniques (endoscopic cholangiography or MRI) [31, 32]. Patients who present with clinical, biochemical and histological features compatible with PSC, but have a normal cholangiogram, are classified as small duct PSC [31, 33]. The histological hallmark of PSC is represented by concentric fibrosis of bile ducts; however, this finding is detected in only 30 to 40% of patients with PSC [31, 34-36]. Onion-skin fibrosis can also be found in secondary sclerosing cholangitis, but the patient reported here had no identified cause to this condition [31].

Autoantibodies are often found in PSC patients, but none of them have enough sensitivity and/or specificity to be considered a diagnostic maker. Autoantibodies regularly described in PSC include ANA (8-77%), SMA (0-83%), and pANCA (26-94%), usually in low titers [12-14, 37].

Description of overlapping between PBC and PSC is very uncommon. After revision of literature, only five reports of PBC/PSC overlapping syndromes were identified [15-18]. MRI was normal in the present patient, leading to the diagnosis of small duct PSC/PBC overlapping. To the best of our knowledge, this is the first report of small duct PSC overlapping with PBC. As she presented positivity to gp210, prognosis is a matter of awareness, even in the presence of small duct PSC, a condition with better prognosis than large duct PSC [38, 39]. We have not enough follow up for evaluating prognosis in this case. After five years, response to treatment has been satisfactory.

In conclusion, we describe a female patient with features of PBC and small duct PSC, a diagnosis that would remain undiscovered if histology was not performed. This finding has important and controversial clinical implications, such as the management of treatment and the need of screening for cholangiocarcinoma [32]. This case is emblematic of how complex diagnosis of liver autoimmune diseases can be, and reinforces the impression that these are polymorphic entities that can express different patterns of autoantibodies and histological findings, making the diagnosis of these conditions particularly challenging.
